# Negative association between ascaris lumbricoides seropositivity and Covid-19 severity: insights from a study in Benin

**DOI:** 10.3389/fimmu.2023.1233082

**Published:** 2023-08-09

**Authors:** Tomabu Adjobimey, Julia Meyer, Anneka Hennenfent, Anick J. Bara, Latifou Lagnika, Bienvenu Kocou, Marius Adjagba, Anatole Laleye, Achim Hoerauf, Marijo Parcina

**Affiliations:** ^1^ Institute of Medical Microbiology, Immunology and Parasitology (IMMIP), University Hospital Bonn, Bonn, Germany; ^2^ Laboratoire de Biologie intégrative pour l’Innovation thérapeutique (BioInov), Faculté des Sciences et Techniques (FAST), Université d’Abomey Calavi, Abomey Calavi, Benin; ^3^ Laboratoire de Cytogénétique, Faculté des Sciences de la Santé (FSS), Université d’Abomey-Calavi, Cotonou, Benin; ^4^ Bonn-Cologne Site, German Center for Infectious Disease Research (DZIF), Bonn, Germany

**Keywords:** *Ascaris lumbricoides*, COVID-19, SARS-CoV-2, healthy donors, neutralizing antibodies, asymptomatic infection

## Abstract

**Introduction:**

The COVID-19 pandemic has had devastating effects worldwide, but the trajectory of the pandemic has been milder in Low-and-Middle-Income Countries (LMICs), including those in Africa. Co-infection with helminths, such as Ascaris lumbricoides, has been suggested as a possible factor contributing to the reduced severity observed in these regions.

**Methods:**

The present study investigated the association between Ascaris-specific antibody levels and COVID-19 severity in 276 SARS-CoV-2-infected individuals in Benin. Participants were categorized into asymptomatic (n=100), mild (n=150), and severe (n=26) groups based on clinical disease severity. Sera were collected and analyzed using ELISA to measure Ascaris and SARS-CoV-2-specific antibodies, while Luminex was used to assess cytokines and SARS-CoV-2-specific neutralizing antibody expression.

**Results and discussion:**

The results demonstrated that asymptomatic SARS-CoV-2 seropositive individuals expressed, on average, 1.7 and 2.2-times higher levels of Ascaris antibodies compared to individuals with mild and severe COVID-19, respectively. This finding suggests an inverse correlation between Ascaris antibody levels and COVID-19 severity. Notably, logistic regression analysis showed that Ascaris seropositivity was significantly associated with a reduced risk of severe COVID-19 (OR = 0.277, p = 0.021). Interestingly, COVID-19 patients with comorbidities such as type 2 diabetes and high blood pressure showed lower expression of Ascaris antibodies. Strikingly, no correlation was observed between Ascaris antibody levels and SARS-CoV-2-specific neutralizing antibodies. On the other hand, individuals seronegative for Ascaris displayed significantly higher levels of systemic pro-inflammatory markers compared to seropositive individuals. These findings suggest that higher expression of Ascaris antibodies is associated with asymptomatic SARS-CoV-2 infections and may contribute to the reduction of the risk to develop severe COVID-19. The beneficial effect of Ascaris seropositivity on COVID-19 outcomes in Benin may be attributed to a decrease in comorbidities and pro-inflammatory markers. These observations provide valuable insights into the milder COVID-19 trajectory observed in Africa and may have implications for future therapeutic strategies.

## 1 Introduction

Since the first cases were reported in Wuhan, China, in November 2019, the COVID-19 pandemic, caused by severe acute respiratory syndrome coronavirus 2 (SARS-CoV-2), has been causing significant morbidity and mortality worldwide and represents one of the most devastating health crises in human history (1). Globally, as of May 26^th,^ 2023, there have been 766 895 075 confirmed cases of COVID-19, including 6 935 889 deaths, reported to the World Health Organisation (WHO) (2). It is well-established that age and pre-existing comorbidities are the main determinants of COVID-19-related mortality (3). Moreover, a discernible trend towards a higher susceptibility to severe disease has been observed in the male population (4). According to the virulence of the variants, patients with SARS-CoV-2 infection can experience a broad spectrum of clinical manifestations, ranging from asymptomatic infection to critical illness. In general, patients with SARS-CoV-2 infection can be grouped into 5 categories, including asymptomatic and pre-symptomatic infection, mild illness, moderate illness, severe illness, and critical illness (5). The asymptomatic and pre-symptomatic group includes patients with no visible sign of the infection (5). Although the percentage of truly asymptomatic SARS-CoV-2 infections varies from one region to another, it is estimated that asymptomatic infections account for at least one-third of the total cases (5). While it remains unclear what percentage of asymptomatic individuals progress to clinical illness, it is well established that asymptomatic and pre-symptomatic individuals contribute to the spread of the virus (6).

Well-equipped intensive care units (ICUs) are necessary to save the lives of severe and critically ill COVID-19 patients. Even with advanced health infrastructure and robust health systems, developed countries have struggled to manage the surge in COVID-19 victims, leading to unprecedented stress levels in ICUs (7–9). In addition to the millions of lives lost due to COVID-19, devastating effects of the pandemic on social interactions, education, and the psychological well-being of people have been reported in the developed world (10–12). In contrast, Sub-Saharan African countries have witnessed a less severe impact of the pandemic (13). Despite having the lowest COVID-19 vaccination rates, these countries have not experienced the predicted scales of mortality and morbidity (14, 15). This led to hypotheses suggesting that exposure to tropical parasites, including helminths, and specific demographic factors could have contributed to the reduced severity of COVID-19 in these regions (16–21). Indeed, infectious diseases do not exist in isolation, and co-existing infections can significantly modulate their impact (22, 23). Particularly, helminths, known for their immune-modulating effects, can shift immune responses from a Th1 to a Th2 type and increase regulatory T-cell activity, which might impact the host’s response to other pathogens, including SARS-CoV-2 (24). The peculiar trajectory of the COVID-19 pandemic in Sub-Saharan Africa, characterized by lower severity despite low vaccination rates, raises questions about the role of such co-infections in modulating SARS-CoV-2 infection outcomes. Notably, Benin, the setting for this study, is characterized by a high prevalence of helminth infections, including ascariasis (25). Ascariasis, a parasitic infection caused by *Ascaris lumbricoides*, is the most common helminth infection worldwide (26). This soil-transmitted helminth parasite affects an estimated 807-1,221 million people, mainly in regions marked by poverty and poor socioeconomic conditions (26). Like most helminths, *Ascaris lumbricoides* is highly prevalent within communities with high poverty and poor socioeconomic status (27).

Despite being generally asymptomatic, the infection involves a complex lifecycle that promotes chronic immune modulation in the host (27). Data on the interplay between helminthic infections and COVID-19 remains limited, with few studies directly investigating this relationship. Furthermore, the implications of these findings for public health, particularly in regions where helminth infections are prevalent, have yet to be fully explored. In light of these observations, a recent study in our group has demonstrated that in contrast to *Plasmodium falciparum*, helminth antigens, including *Ascaris*, suppress the activation of SARS-CoV-2 specific CD4+ T cells of convalescent COVID-19 patients and might potentially impact the course of a SARS-CoV-2 infection (28). Building on these findings, the present study aims to investigate the relationship between *Ascaris* antibody expression and COVID-19 severity, providing critical insights into the role of helminth co-infection in the risks of developing severe SARS-CoV-2 infections. The results of the study could have far-reaching implications for our understanding of the multifactorial determinants of COVID-19 severity and inform public health strategies in regions where helminth infections are endemic.

## 2 Materials and methods

### 2.1 Participants, sample collection, and ethics

**Figure 1:**
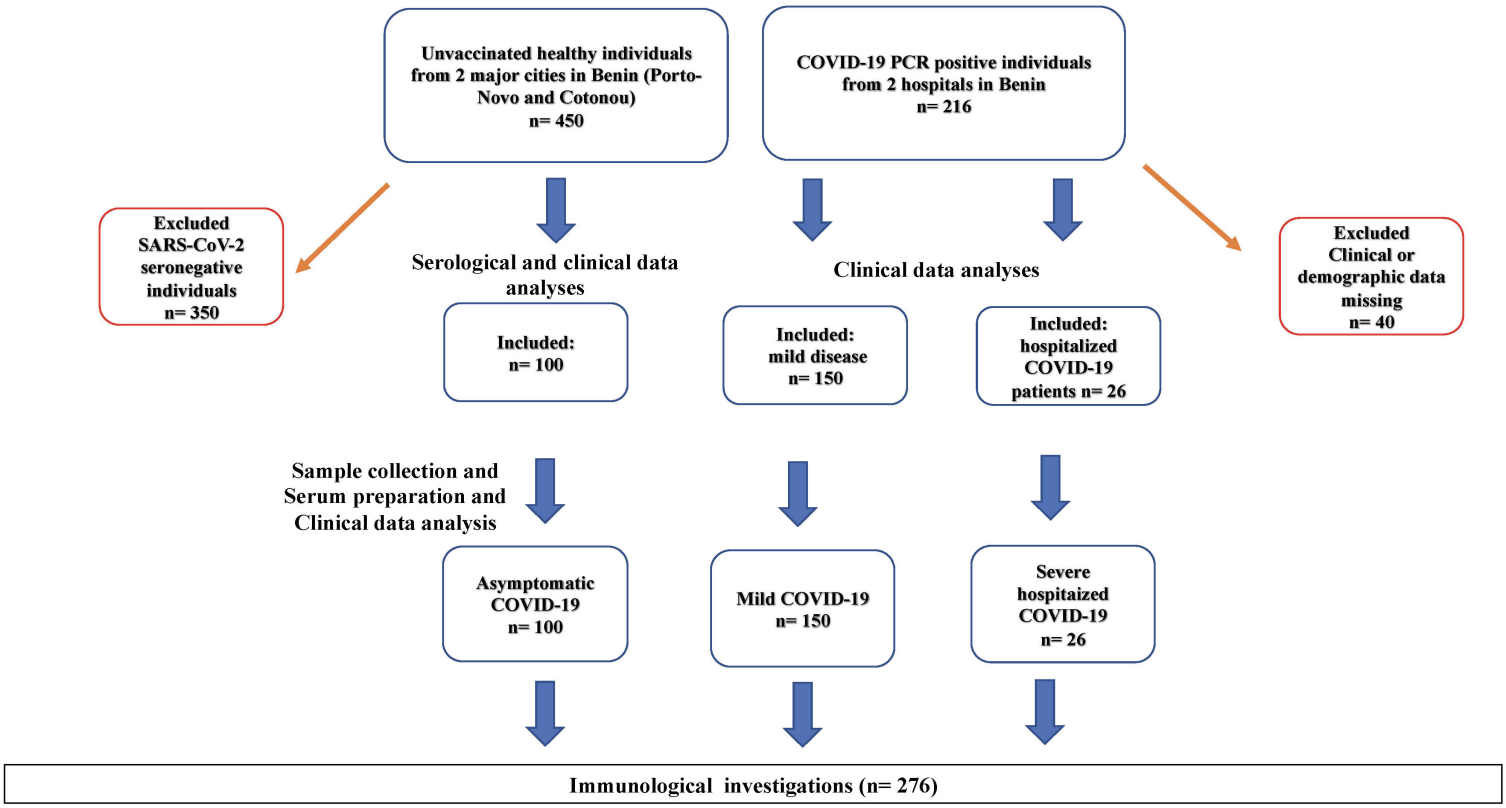
Participant Selection Flow Chart. SARS-CoV-2 seropositivity tests were conducted using rapid immunochromatographic antibody tests (NADAL) and confirmed by ELISA. 100 asymptomatic SARS-CoV-2 infected individuals were identified from a cohort of randomly selected healthy volunteers (n=450) in churches, universities, and large family houses in two major cities of Benin (Cotonou and Porto-Novo). Furthermore, 176 COVID-19 patients, including 150 with mild disease and 26 hospitalized with severe COVID-19, were recruited from a cohort of 360 COVID-19 patients admitted to official COVID-19 care centers at the University Hospital of Cotonou (Centre National Hospitalier Universitaire Hubert Koutougou MAGA, or CNHU-HKM) and the Hospital of Abomey-Calavi. Seronegative healthy individuals (n=350) and COVID-19 patients who opted not to provide demographic or clinical data (n=40) were excluded from the analyses.

A total of 666 individuals, including 450 healthy donors and 216 COVID-19 patients, were identified in two major cities of Benin. COVID-19 patients were recruited from official COVID-19 care centers at the University Hospital of Cotonou (Centre National Hospitalier Universitaire Hubert Koutougou MAGA, or CNHU-HKM) and the Regional Hospital of Abomey-Calavi. The survey was conducted between June 2021 and December 2022 by teams from the Cytogenetic and Biochemistry and Molecular Biology Units of the University of Abomey-Calavi in Benin. Healthy donors were screened for SARS-CoV-2 specific antibodies using the NADAL^®^ COVID-19 IgG/IgM rapid immunochromatographic test. The clinical files of COVID-19 patients were accessed to obtain clinical and demographic data. Of the 216 COVID-19 patients, 40 were excluded from the analyses due to insufficient clinical information. Out of the 450 asymptomatic donors, 100 were seropositive for SARS-CoV-2 and were included in the study as asymptomatic SARS-CoV-2 infected individuals (**Figure 1**). Finally, a total of 276 adult participants including 176 COVID-19 patients and 100 asymptomatic SARS-CoV-2 positive individuals were included in the study. All COVID-19 diagnoses were confirmed via PCR testing. Among the COVID-19 patients, 150 developed mild symptoms, while 26 had severe COVID-19 requiring hospitalization. The group of 100 asymptomatic individuals consisted of SARS-CoV-2 seropositive individuals who remained symptom-free before and at least14 days after sampling and had no history of COVID-19 infection or vaccination. Disease severity data were collected under clinical supervision. Participants provided informed consent, and ethical clearance was obtained from the Ethics Committee for Biomedical Research of the University of Parakou in Benin (Reference N°: 0458/CLERB-UP/P/SP/R/SA). The study adhered to the guidelines and regulations of the Benin Ministry of Health (Authorization N°: 3856/MS/DC/SGM/DRFMT/SRAO/SA). Venous blood samples were collected using the S-Monovette SERUM GEL blood collection system (Sarstedt AG, Nümbrecht, Germany). Sera were obtained by centrifugation, and two aliquots were prepared. The first aliquot was used onsite for serological and parasitological investigations. The second was frozen at -20°C and shipped to Germany for ELISA and Luminex assays. Ethics approval for *in vitro* experiments in Germany was obtained from the Ethical board of the University Hospital Bonn (Lfd.Nr.439/20). The study design and sample collection procedures are summarized in **Figure 1**.

### 2.2 Serological testing for SARS-CoV-2 antibodies with the NADAL^®^ COVID-19 IgG/IgM rapid test in healthy volunteers

The NADAL^®^ COVID-19 IgG/IgM rapid immunochromatographic test was used onsite to detect SARS-CoV-2-specific IgM and IgG antibodies against the spike protein (S protein) or the nucleocapsid protein (N protein) in 450 healthy volunteers. These individuals were recruited in churches, universities, and family houses in two major cities of Benin, Cotonou, and Porto-Novo. Eligibility for the study required participants to be asymptomatic at the time of testing, have no known history of COVID-19 infection, and not be vaccinated against the disease. Each participant provided a blood sample, which was applied to the test strip. The test was conducted following the manufacturer’s instructions using the provided buffer. A result was considered negative if only the control line appeared. If both the control line and the IgM or IgG antibody line appeared, the result was deemed positive, indicating the presence of SARS-CoV-2 specific IgM or IgG antibodies, respectively. Results were recorded, and blood samples were collected from seropositive individuals for further analysis. All participants’ information was anonymized to ensure privacy and confidentiality.

### 2.3 Quantification of SARS-CoV-2 specific antibodies

Enzyme-Linked Immunosorbent Assays (ELISAs) were conducted at the Institute of Medical Microbiology, Immunology, and Parasitology of the University Clinic of Bonn, Germany. We utilized the Euroimmun SARS-CoV-2 IgG/IgA ELISA kit (Euroimmun, Lübeck, Germany) to detect the levels of SARS-CoV-2 specific IgG and IgA, adhering to the manufacturer’s instructions and following the protocol previously described (29). In brief, serum samples were diluted 1:101 using the provided sample buffer and then incubated at 37° C for 60 minutes in a 96-well microtiter plate. The washing and incubation cycles were automated using the predesigned program of Euroimmun’s Analyzer I. The optical density (OD) was measured at 450 nm. The expressions of SARS-CoV-2 specific immunoglobulin G and A were calculated, and the results were interpreted in accordance with the manufacturer’s protocol.

### 2.4 Detection of *Ascaris lumbricoides*-specific IgG

*Ascaris lumbricoides* IgG ELISA Kit (DRG Instruments GmbH, Marburg, Germany) was used to detect specific IgG immunoglobulins in sera of COVID-19 patients and controls from Benin. The procedure was manually executed following the manufacturer’s protocol. In brief, the samples were diluted (1:101) using the appropriate sample buffer. A volume of 100 µl of samples, standards, and controls was added to each well of the pre-coated 96-well plate and incubated for 1 hour at 37°C. Following this, the wells were washed three times with 300 µl of washing solution. Subsequently, 100 µl of conjugate was added and incubated for 30 minutes at room temperature. After another round of washing, 100 µl of substrate (TMB) was added and incubated for 15 minutes at room temperature in the dark. The reaction was stopped by adding 100 µl of the provided stop solution. The optical density (OD) was measured at 450/620nm. The absorbance was then converted into units (Index) following the manufacturer’s protocol using this formula: (sample absorbance value x 10)/(Cut-Off) = DRG Units (DU). The results were interpreted as per the manufacturer’s guidelines: >11 DU was considered positive, 9-11 DU as equivocal, and <9 DU as negative. The diagnostic specificity is reported at 95%, and sensitivity at 100%. It’s important to note that cross-reactivity with antibodies against Toxocara canis, Trichinella, Fasciola, Filaria, and Strongyloides cannot be excluded.

### 2.5 Quantification of neutralizing antibody levels

To estimate the levels of neutralizing SARS-CoV-2 variant-specific antibodies, the SARS-CoV-2 Variants Neutralizing Antibody 6-Plex ProcartaPlex Panel (ThermoFisher Scientific) was used according to the manufacturer’s instructions. The test is a commercially available version of the previously described SARS-CoV-2 surrogate virus neutralization test based on antibody-mediated ACE2–spike protein–protein interaction blockage (30). The assay was seen to be as specific but more sensitive than cell-based assays (30). The assay is designed to detect the level of SARS-CoV-2 neutralizing antibodies in serum and plasma and allows a direct comparison of neutralizing antibody expressions towards five variants of SARS-CoV-2, including wild-type and variants B.1.1.7 (α), B.1.351 (β), P1 (γ), B.1.617.2 (δ), and B.1.1.529 (o). Briefly, 50 μL of capture beads were added to each well of the provided 96 microtiter plate and washed with 1X washing solution. 25 μL of prediluted (1:1) samples were added to the corresponding wells. Positive and negative controls were included as per the manufacturer’s recommendations. The plates were then sealed and incubated at room temperature with moderate shaking (500 rpm/minute) for 120 minutes. After 2 washes, 25 μL of prediluted 1X Detection Antibody mixture was added to each well. Plated were then incubated at room temperature for 30 additional minutes. After an additional 2 times wash, 50 μL of SA-PE solution was added to each well, and plates were further inculcated again for 30 minutes. After a final round of 2 washes, 120 μL of Reading Buffer was added to each well. Plates were then incubated at room temperature for 5 minutes and run on the MagPix Luminex instrument. The results were analyzed using the ProcartaPlex analyst application (ThermoFisher Scientific) and according to the provided neutralization (%) equation.

### 2.6 Quantification of systemic cytokine and chemokine expression

To estimate the systemic cytokine expression, Cytokine Storm 21-Plex Human ProcartaPlex Panel (ThermoFisher Scientific) was used according to the manufacturer’s instructions. The assay covers 21 cytokines and markers related to cytokine release syndrome (CRS), including G-CSF (CSF-3), GM-CSF, IFN alpha, IFN gamma, IL-1 beta, IL-2, IL-4, IL-5, IL-6, IL-8 (CXCL8), IL-10, IL-12p70, IL-13, IL-17A (CTLA-8), IL-18, IP-10 (CXCL10), MCP-1 (CCL2), MIP-1 alpha (CCL3), MIP-1 beta (CCL4), TNF alpha, and TNF beta. Briefly, 50 μL of capture beads were added to each well of the provided 96 microtiter plate and washed with 1X washing solution. 25 μL of prediluted (1:1) samples were added to the corresponding wells. Positive and negative controls were included as per the manufacturer’s recommendations. The plates were then sealed and incubated at room temperature with moderate shaking (500 rpm/minute) for 120 minutes. After 2 washes, 25 μL of prediluted 1X Detection Antibody mixture was added to each well. Plates were then incubated at room temperature for 30 additional minutes. After an additional 2 times wash, 50 μL of SA-PE solution was added to each well, and plates were further inculcated again for 30 minutes. After a final round of 2 washes, 120 μL of Reading Buffer was added to each well. Plates were then incubated at room temperature for 5 minutes and run on the MagPix Luminex instrument. The results were analyzed using the ProcartaPlex Analyst application (ThermoFisher, Scientific).

### 2.7 Statistics

All graphs were generated using GraphPad Prism 9 (La Jolla, CA, USA). Kruskal-Wallis test, followed by Dunn’s posthoc test, was used to compare age, BMI, and *Ascaris* antibody expression across different clinical groups. Distribution of the data was analyzed using both Shapiro-Wilk test and visual inspection of Q-Q plots. Nonparametric Spearman correlation was performed to analyze relationships between age, BMI, and *Ascaris* antibody expression. Mann-Whitney-test was used to compare cytokine and antibody expressions between *Ascaris* seronegative and seropositive groups. Multiple logistic regression analysis to determine the impacts of different factors, including age, obesity (BMI over 30), *Ascaris* seropositivity, high blood pressure (BP), type 2 diabetes (Diabetes), and sex on the risk of developing Severe COVID-19 (Hospitalization) was done using R. A p-value of less than 0.05 was considered statistically significant.

## 3 Results

### 3.1 Demographic and clinical data

The demographic and clinical data of the study participants are summarized in **Table 1**. Of the total of 276 volunteers investigated, the majority exhibited mild COVID-19 symptoms (n=150), followed by asymptomatic patients (n=100), and a minority of severe cases (n=26). The mean age differed across severity levels. Asymptomatic patients had a mean age of 42.93 ± 13.36 years, while mild patients had a mean age of 41.84 ± 13.81 years. In contrast, the average age among severe cases was notably higher, at 53.73 ± 11.88 years. This suggests that older age might be associated with a higher risk of severe COVID-19. The average Body Mass Index (BMI) also differed across the groups. Asymptomatic patients had a mean BMI of 24.83 ± 5.33, while mild patients had a mean BMI of 25.22 ± 5.57. The severe COVID-19 group had a slightly higher mean BMI of 26.43 ± 4.50. This hints at a potential association between higher BMI and increased COVID-19 severity. Regarding gender distribution among the different disease severity levels, a larger proportion of females were found among the asymptomatic (62%, n=62) and mild (72.67%, n=109) cases. A higher proportion of males (57.69%, n=15) presented severe cases. This confirmed gender disparity in COVID-19 severity.

**Table 1:** Demographic and clinical characteristics by COVID-19 severity level.

	Asymptomatic	Mild	Severe	Total
N	100	150	26	276
Age (±SD)	42.93 ± 13.36	41.84 ± 13.81	53.73 ± 11.88	43.35 ± 13.86
BMI (± SD)	24.83 ± 5.33	25.22 ± 5.57	26.43 ± 4.50	25.20 ± 5.39
Female (n)	62% (62)	72.67% (109)	42.31% (11)	65.94% (182)
%Male (n)	38% (38)	27.33% (41)	57.69% (15)	34.06% (94)
%T2D (n)	4% (4)	8% (11)	30% (8)	8.33% (23)
%HBP (n)	6% (6)	11% (17)	50% (13)	13.04% (36)
%RI (n)	0% (0)	4% (6)	0(0%)	2.17% (6)

This table shows demographic and clinical characteristics across different COVID-19 severity levels: asymptomatic, mild, and severe. Each category displays the total number of individuals, mean age and BMI with standard deviation, and the percentage (and number) of individuals who are female, male, and those with comorbidities such as type 2 diabetes (T2D), high blood pressure (HBP), and renal insufficiency (RI).

For each category, the number (N) of individuals included to the study is provided, the mean age and body mass index (BMI) with their standard deviations (SD), and the percentage (and absolute number) of individuals who are female, male, and those diagnosed with type 2 diabetes (T2D), high blood pressure (HBP), and renal insufficiency (RI). The total column provides the corresponding data for the overall sample.

Three comorbidities were recorded: Type 2 Diabetes (T2D), High Blood Pressure (HBP), and Renal Insufficiency (RI). T2D and HBP were more prevalent in severe cases (30%, n=8, and 50%, n=13, respectively), suggesting a potential association between these conditions and disease severity. Conversely, RI was only observed in the mild cases (4%, n=6) and was not recorded in either the asymptomatic or severe groups (**Table 1**).

### 3.2 *Ascaris* antibody expression negatively correlated with COVID-19 severity

To identify factors associated with the severity of COVID-19, a multiple logistic regression analysis was performed. This examined the relationships between demographic and clinical features and the severity of COVID-19. The findings are displayed in **Table 2**. A statistically significant association was found between age and the severity of COVID-19, with an odds ratio (OR) of 1.077 and a statistically significant p-value of 0.007. This indicates that the odds of severe COVID-19 increase by approximately 7.7% for each additional year of age. Interestingly, a significant negative association was observed between *Ascaris* seropositivity and the severity of COVID-19. The OR of this association was 0.277 with a p-value of 0.021, suggesting that individuals who tested positive for *Ascaris* have approximately 72.3% lower odds of experiencing severe COVID-19 symptoms. The presence of high blood pressure (OR = 0.287, p = 0.131) and diabetes (OR = 0.340, p = 0.324) were found to be associated with the severity of COVID-19, although these associations were not statistically significant (p > 0.05). Despite the lack of statistical significance, the ORs suggest that individuals with these conditions may have higher odds of developing severe symptoms, indicating potential risk factors. Contrary to common assumptions, obesity demonstrated a negligible correlation in this study (OR = 0.865, p = 0.839), suggesting that in this sample, obesity was not a significant risk factor for severe COVID-19. Few renal insufficiency (RI) cases were recorded and included in the analysis. However, this variable’s estimates were not significantly associated with the outcome (p = 0.992). Lastly, the analysis revealed a significant positive correlation between male sex and severe COVID-19 (OR = 2.685, p = 0.030). This suggests that males are approximately 2.7 times more likely to experience severe COVID-19 symptoms compared to females (**Table 2**).

**Table 2:** Age, *ascaris* seropositivity, and sex as significant predictors of COVID-19 severity in benin.

Variable	Estimate	Standard Error	Odds Ratio	Adjusted p value	95% Confidence Interval
*(Intercept)*	*-5.653*	*1.248*	*0.004*	*< .001*	*0.000 - 0.040*
Age	0.074	0.028	1.077	0.007	1.020 - 1.137
Ascaris seropositive (1)	-1.283	0.557	0.277	0.021	0.093 - 0.825
HBP (1)	-1.248	0.827	0.287	0.131	0.057 - 1.452
T2D (1)	-1.080	1.096	0.340	0.324	0.040 - 2.909
Obesity (1)	-0.145	0.716	0.865	0.839	0.212 - 3.520
RI (1)	-15.974	1509.090	1.15x10^-7^	0.992	N/A
Sex (1)	0.988	0.456	2.685	0.030	1.099 - 6.559

This table presents the results of a multiple logistic regression analysis investigating factors associated with the severity of COVID-19. Variables include age, Ascaris seropositivity, high blood pressure, diabetes, obesity, sex, and renal insufficiency, with their corresponding estimates, standard errors, odds ratios, adjusted p-values, and 95% confidence intervals.

The presence of comorbidities including high blood pressure (HBP), diabetes (T2D), renal insufficiency (RI) as well as Ascaris seropositivity and male sex are coded as 1. All p values are age-adjusted. The intercept corresponds to the log-odds of the outcome when all variables are absent. N/A = not applicable.

### 3.3 Asymptomatic SARS-CoV-2 infected individuals expressed higher levels of *Ascaris* specific antibodies

**Figure 2:**
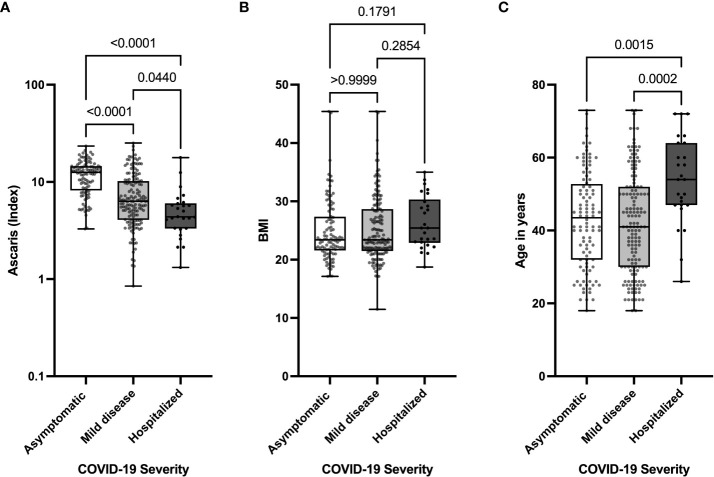
Lower Levels of *Ascaris*-specific Antibodies in Hospitalized COVID-19 Patients. Box and whisker plots depict the distribution of the *Ascaris* antibody index **(A)**, BMI **(B)**, and age **(C)** in three groups, including asymptomatic (clear), mild (light grey), and severe (dark grey) COVID-19 patients. The dots represent individual patients. Bars indicate the means with the minimum and maximum values.

To further analyze the effects of *Ascaris* antibody expression on COVID-19 severity, we compared the expression of *Ascaris*-specific antibodies in asymptomatic, mild, and hospitalized COVID-19 patients. The data indicate that asymptomatic SARS-CoV-2 seropositive individuals expressed on average 1.7X more *Ascaris* antibodies compared to COVID-19 patients with mild disease and 2.2X more *Ascaris* antibodies when compared to hospitalized COVID-19 patients (**Figure 2A**). To find how BMI and age affected COVID-19 severity, we compared age BMI expression and age in the 3 COVID-19 severity groups. The results indicate that, while no significant difference was seen in the BMI (**Figure 2B**), hospitalized COVID-19 patients were on average 10 years older than their counterparts with mild COVID-19 and asymptomatic infection. Interestingly, no significant difference was seen when the ages of asymptomatic and mild COVID-19 patients were compared (**Figure 2C**). These data suggest that advanced age is associated with severe COVID-19, whereas *Ascaris* antibody expression is associated with asymptomatic SARS-CoV-2 infection and a lesser extent to mild COVID-19.

### 3.4 Lower expression of *Ascaris* antibody in COVID-19 patients with comorbidities

**Figure 3:**
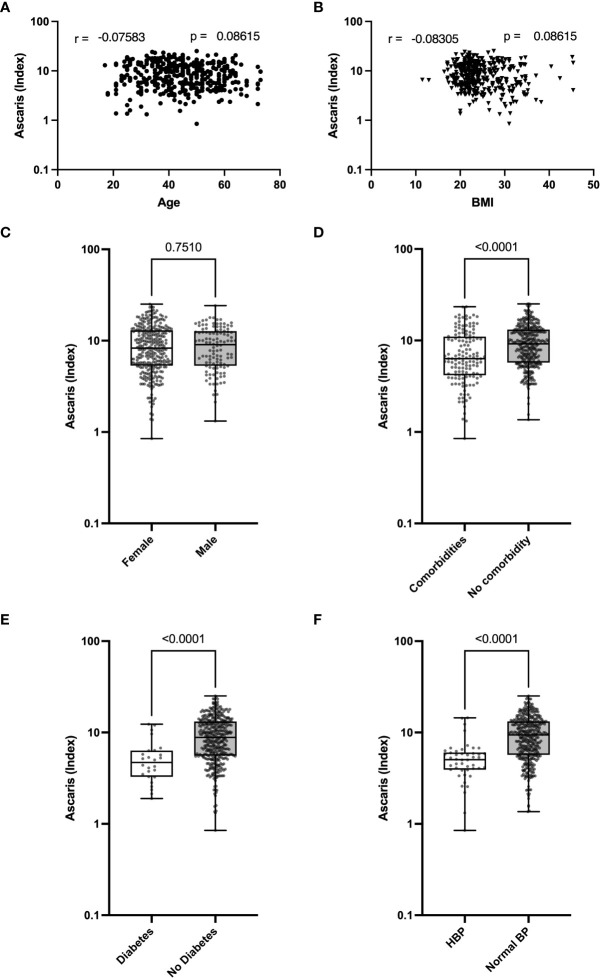
Lower Expression of *Ascaris* Antibodies in COVID-19 Patients with High Blood Pressure and T2D. Graphs represent correlation analyses of *Ascaris* antibody expression according to age **(A)** and BMI **(B)**. Box and whisker plots depict the distribution of the *Ascaris* antibody index in males and females **(C)**, in COVID-19 patients with or without comorbidities **(D)**, in patients with or without T2D **(E)**, and in patients with or without HBP **(F)**. The dots represent individual patients. Bars indicate the means with the minimum and maximum values.

While *Ascaris* infection can directly affect COVID-19 severity, it is possible that the presence of the parasite is related to age or impacts COVID-19-relevant comorbidities such as high blood pressure, obesity, diabetes, or renal insufficiency and thus indirectly modulate the severity of COVID-19. To determine if a link exists between *Ascaris* antibody expression and COVID-19-relevant comorbidities, including age, BMI, sex, and high blood pressure, we correlated the expression of *Ascaris* antibody with age and BMI. In addition, we analyzed the expression of *Ascaris*-specific antibodies in the different clinical and demographic groups. The data suggest no noticeable correlation between *Ascaris* antibody expression and age or BMI (**Figures 3A, B**). In addition, no significant difference was seen in the expression of *Ascaris* antibodies in male and female COVID-19 patients (**Figure 3C**). In contrast, *Ascaris* antibody expression in COVID-19 patients with at least one comorbidity, including diabetes and high blood pressure, was significantly lower when compared to patients without these comorbidities (**Figures 3D–F**). These findings suggest that while there is no association between *Ascaris* antibody expression and age, BMI, or sex in COVID-19 patients, *Ascaris* antibody expression is generally lower in COVID-19 patients exhibiting high blood pressure and/or diabetes.

### 3.5 No difference in SARS-CoV-2 neutralizing antibody expression in *Ascaris* seropositive and Negative COVID-19 patients

**Figure 4:**
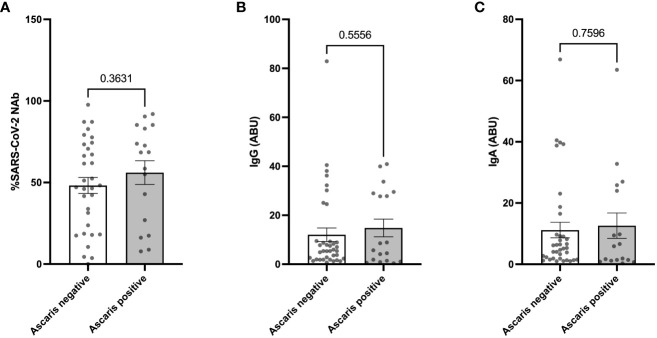
No Difference in SARS-CoV-2 Specific and Neutralizing Antibody Expression in *Ascaris* Seropositive and Negative Individuals. Graphs represent the relative expression of neutralizing antibodies **(A)**, anti-spike IgG **(B)**, and IgA **(C)** in sera of *Ascaris* seropositive and negative individuals. Bars represent the mean percentages of antibody expression ± SEM. Dots represent antibody expression in individual patients.

In our subsequent analysis, we sought to determine whether the observed milder COVID-19 symptoms in *Ascaris* seropositive individuals corresponded with a heightened expression of SARS-CoV-2 specific antibodies. Interestingly, the data revealed a comparable expression of SARS-CoV-2 specific neutralizing antibodies (NAbs) in both *Ascaris* seropositive and seronegative individuals. There was no significant difference in the expression of SARS-CoV-2 NAbs among Ascaris seropositive individuals, as evidenced by the results of the Student’s T test (**Figure 4A**). This trend remained consistent when we measured the levels of anti-SARS-CoV-2-spike IgG and IgA antibodies (**Figures 4B, C**). These findings suggest that *Ascaris* co-infection does not influence the capacity of SARS-CoV-2 infected individuals to generate an effective humoral immune response against the virus.

### 3.6 Higher systemic pro-inflammatory cytokine expression in *Ascaris*-negative COVID-19 patients

**Figure 5:**
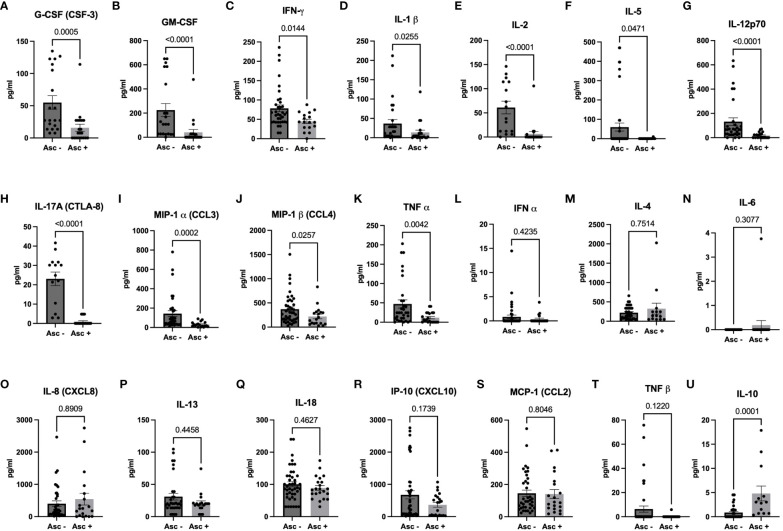
Higher Expression of Pro-inflammatory Markers in Sera of *Ascaris* Seronegative Individuals. The graphs represent the relative expression of G-CSF **(A)**, GM-CSF **(B)**, IFN-γ **(C)**, IL-1β **(D)**, IL-2 **(E)**, IL-5 **(F)**, IL-12p70 **(G)**, IL-17A **(H)**, MIP-1α **(I)**, MIP-1β **(J)**, TNFα **(K)**, IFNα **(L)**, IL-4 **(M)**, IL-6 **(N)**, IL-8 **(O)**, IL-13 **(P)**, IL-18 **(Q)**, IP-10 **(R)**, MCP-1 **(S)**, TNFβ **(T)**, and IL-10 **(U)** in the sera of individuals who are either seropositive (light grey bars) or seronegative (dark grey bars) for *Ascaris*. Bars indicate the mean percentages of cytokine expression ± SEM, while the dots represent individual donors.

Since helminth seropositivity had no impact on the expression of SARS-CoV-2 specific neutralizing antibodies (NAbs), we further examined the systemic cytokine expression in SARS-CoV-2 infected patients, comparing individuals who were seropositive for *Ascaris* with those who were seronegative. Interestingly, our analysis revealed a lower expression of several cytokine storm-related cytokines in *Ascaris* seropositive individuals. Out of the 21 serum markers tested, 11 showed significantly lower levels in *Ascaris* seropositive individuals (**Figures 5A–K**), including pleiotropic myeloid growth factors such as G-CSF and CM-CSF, as well as Th1, Th2, and Th17-related cytokines and chemokines. However, no significant differences were observed for nine cytokines, including IFN-alpha, IL-4, IL-6, IL-8, IL-13, IL-18, IP10, MCP-1, and TNF beta (**Figures 5L–T**). Notably, systemic IL-10 expression was significantly higher in *Ascaris* seropositive individuals compared to their seronegative counterparts (**Figure 5U**).

## 4 Discussion

Our study described the characteristics and clinical features of 276 SARS-CoV-2-infected individuals in Benin. The analysis of the clinical and demographic data in our study confirms initial findings suggesting that factors such as older age, higher BMI, male gender, and presence of certain comorbidities (T2D and HBP) are associated with increased COVID-19 severity in this sample of patients from Benin (31–37). However, these trends were not significant when these comorbidities were analyzed in a multiple regression considering *Ascaris* seropositivity and age. The main finding in this study is the negative association between *Ascaris lumbricoides* seroprevalence and COVID-19 severity. The negative correlation between *Ascaris* antibody expression and COVID-19 severity suggests that recent and current *Ascaris* infections may have reduced the risk of severe COVID-19. This result is in line with the data of an observational study in Ethiopia by Wolday et al., where it was suggested that co-infection with enteric parasites, including protozoa and helminths, is associated with lower odds of developing severe COVID-19 (38).

Further extending the scope of our research, the relationship between *Ascaris* antibody expression and COVID-19 severity can be contextualized through the lens of early-life environmental exposures. Indeed, it is well-established that nutritional and microbial exposures in infancy play an influential role in shaping the development of inflammation in adulthood (39). This relationship may be particularly pertinent in low-income countries, where exposure to *Ascaris lumbricoides* and other parasitic infections are often more common due to limited sanitation and healthcare resources (26). These environments can potentially result in a different baseline of immune responses, which may contribute to the variable disease outcomes observed during the COVID-19 pandemic. As suggested in McDade’s work, these early environments help to shape the regulation of inflammation, thereby modulating responses to inflammatory stimuli later in life (40). The presence of *Ascaris lumbricoides* could modulate the immune system and decrease the severity of SARS-CoV-2 infection. This could occur through the downregulation of systemic pro-inflammatory markers, as we observed lower levels of these markers in individuals seropositive for *Ascaris*. This hypothesis is supported by data from Alhamwe et al., who, in a rodent model of repeated intranasal administration of *Acinetobacter lwoffii*, found that protection was associated with IL-6-induced IL-10 production in CD4+ T-cells (41). Further supporting this, Zakzuk et al. reporting on the expression of *Ascaris* and house dust mite (HDM)-specific IgE, emphasized the modification of histone acetylation levels at key type-2 immune genes in humans by nematode infection and HDM allergens (42). The relevance of epigenetics for immune responses is particularly notable in light of the review article by Acevedo et al., which discusses the potential impacts of specific nutritional components on epigenetic patterns related to allergies (43). While our study did not directly assess epigenetic changes, it is plausible that the inverse relationship we observed between *Ascaris* antibody levels and COVID-19 severity is supported by similar mechanisms, opening new avenues for future investigations.

Helminth infections, including *Ascaris*, have been linked to Th2 polarized immune responses with prominent IL-4 and IL-5 expression that typically contrasts with the Th1 immune responses associated with viral and bacterial infections (44–47).

The induction of Th2 cytokines by *Ascaris* can, however, not justify the mild course of COVID-19 in co-infected individuals. Indeed, emerging data suggest that poor prognostic during COVID-19 is linked with the production of Th2 cytokines (48). A recent study revealed an unexpectedly elevated count of activated Th2 cells in severe COVID-19 patients (49). The same authors showed that individuals who did not survive COVID-19 exhibited a higher presence of senescent Th2 cells (49). At first glance, these observations imply that helminth infections, also known to induce Th2 responses, may exacerbate COVID-19. In view of these data, several authors have brought attention to the potential adverse associations between severe COVID-19 cases and co-infections with helminths in regions where these parasitic infections are endemic (21, 50). However, emerging evidence contradicts this hypothesis and suggests that helminth co-infection, in addition to the classical Th2 response, can induce a range of immune regulatory mechanisms that can potentially mitigate COVID-19 severity (20, 28). This hypothesis is supported by previous findings describing helminths and their secretory products as potent regulators of the immune system through the induction of regulatory cytokines, T cells, and/or alternatively activated macrophages (AAMs) which were found to directly slow down allergen-specific Th2 responses (51). While our study did not address the expression of regulatory cells, the higher expression of IL-10 detected in *Ascaris*-seropositive individuals indicates that such processes might be implicated. Indeed, AAMs and regulatory T-cell subsets are renowned for producing large amounts of IL-10 to maintain immune homeostasis and control host-microorganism interactions (52–55). This is particularly interesting in light of a recent study conducted in Ghana, which found that symptomatic COVID-19 patients generally had significantly higher cytokine levels compared to asymptomatic cases (56). This study suggests that the immune response, as indicated by cytokine levels, may play a role in the severity of symptoms (56). Although this study does not distinguish between helminth-positive and -negative COVID-19 patients, and it does not include patients with severe COVID-19, our data are largely consistent with the cytokine profile they described. Taking into account both their findings and ours, it seems reasonable to suggest a correlation between the elevated expression of pro-inflammatory cytokines and increased COVID-19 severity.

The negative correlation observed in our study between high blood pressure, diabetes, and *Ascaris* antibody expression may suggests an additional pathway potentially mitigating COVID-19 severity. Indeed, high blood pressure and diabetes are known risk factors for severe COVID-19 outcomes (57, 58). Our findings suggest that *Ascaris* infection may reduce the occurrence of these comorbidities and may thereby indirectly affect the occurrence of severe COVID-19 in helminth endemic regions. These data align with several findings suggesting that helminth infections generally reduce the frequency of metabolic and inflammatory diseases (59). Recent studies in rodent models have demonstrated that helminth infections and helminth-derived products protect against type 1 and type 2 diabetes (60). It was also observed that several helminth infections could reshape the gut microbiota of hosts and ultimately counter the pathogenesis of obesity and associated metabolic syndromes (61). Our results suggest the notion that *Ascaris* infection may have a broader influence on the health status of individuals beyond a direct impact on COVID-19 severity. However, more comprehensive studies including molecular and cellular analyses are needed to confirm these findings. In addition to its potential direct effects on viral infections like SARS-CoV-2, the presence of *Ascaris lumbricoides* infection and subsequent seropositivity may also be an indicator of a broader hygiene status that might correlate with an overall higher potential for immune regulation and immune resilience. Indeed, soil-transmitted helminth infections, including *Ascaris lumbricoides*, generally correlate with poor hygiene standards (62–64).

An alternative explanation could, however, be that individuals with diabetes or high blood pressure are less likely to be infected with *Ascaris* or may mount a less robust immune response to *Ascaris* infection, resulting in lower antibody levels. While this remains a plausible explanation, there is currently no data to support this hypothesis.

Another possible mechanism for COVID-19 risk reduction by *Ascaris* co-infections could be a modulation of SARS-CoV-2-specific antibody expression and neutralization potential. Indeed, several studies have demonstrated that neutralizing antibody levels correlate with COVID-19 severity and lung injury (65, 66). To test the effect of *Ascaris* confections on COVID-19 severity, we measured SARS-CoV-2 neutralizing antibody expression in *Ascaris* seropositive and negative individuals. Our data showed no significant difference in antibody expression between the two groups, suggesting no impact of *Ascaris* infection on humoral immune responses to SARS-CoV-2.

In line with previous reports, our data also suggested as mentioned above, that older individuals are at higher risk to develop severe COVID-19. The importance of age in COVID-19 severity might be associated, as suggested elsewhere, with a greater prevalence of comorbidities in older patients (67–71). Our data could, however, not identify a significant correlation between *Ascaris* antibody expression and age. In the same line, no difference was seen in the expression of *Ascaris* antibodies when comparing male and female SARS-CoV-2 infected individuals. Even though no significant difference was seen in the BMI of the different COVID-19 severity groups, the mean BMI was higher in hospitalized COVID-19 patients compared to individuals with asymptomatic and mild SARS-CoV-2 infections. Noticeable was the high variability seen in the BMI of asymptomatic individuals and COVID-19 patients with mild symptoms. These data suggest that other factors might have modulated the impact of high BMI on COVID-19 severity in Benin. Interestingly, no noticeable correlation was seen between BMI and *Ascaris* antibody expression. Similarly, no difference was seen in *Ascaris* antibody expression in males and females. Our data further revealed lower expression of *Ascaris* antibody expression in COVID-19 patients with high blood pressure and diabetes while nuancing the importance of these comorbidities as risk factors for severe COVID-19 in helminth endemic regions. These data further suggest that while *Ascaris* infection affects the relevance of certain comorbidities for COVID-19 severity, the effect of age and sex is not impacted.

Our data on systemic cytokines and chemokines revealed a lower expression of pro-inflammatory markers in *Ascaris* seropositive individuals compared to their seronegative counterparts. These data support initially suggested mechanisms indicating that parasite-driven immunomodulatory responses may dampen hyper-inflammation associated with severe COVID-19 (28). Similar mechanisms were described in sepsis and metabolic diseases, including diabetes and metabolic syndrome, where helminths and their Excretory/secretory (ES) products were reported to trigger an immune-regulated milieu that inhibits harmful inflammatory processes (72–77). The potent systemic effect of an intestinal helminth infection was elegantly demonstrated in mouse models through the effective prevention of spontaneous type 1 diabetes (T1D) in non-obese diabetic (NOD) mice following *H. polygyrus* inoculation *(* 78, 79). Consistent with previous reports, IL-10 expression was significantly lower in *Ascaris* seronegative patients suggesting a role for this cytokine in the helminth-mediated mitigation of COVID-19 severity (28).

While our study provides compelling evidence suggesting a potential protective role of *Ascaris lumbricoides* infections against severe COVID-19 outcomes, it is important to acknowledge its limitations. First, the use of antibody expression as a marker of *Ascaris* infection may not accurately reflect current infection status, as antibodies can persist after the infection has been cleared (80). Third, our samples were drawn from specific locations in Benin, which may limit the generalizability of our findings to other populations. Ongoing studies in our group aim to address these limitations by using a longitudinal design and including more diverse samples.

Conclusively, our study yielded significant initial insights into the direct and indirect impacts of *Ascaris* infections on the risk of severe COVID-19, affirming our hypothesis that helminth co-infections played a major role in preventing severe outcomes in African countries during the COVID-19 pandemic. These findings hold promising therapeutic implications, overthought further research is required to comprehensively elucidate the underlying mechanisms and evaluate potential applications.

## Data availability statement

The original contributions presented in the study are included in the article/supplementary material. Further inquiries can be directed to the corresponding author.

## Ethics statement

The studies involving human participants were reviewed and approved by Ethics Committee for Biomedical Research of theUniversity of Parakou (Reference N°: 0458/CLERB-UP/P/SP/R/SA). The patients/participants provided theirwritten informed consent to participate in this study.

## Author contributions

TA and AHo conceived the study and were in charge of overall coordination. MP supervised all clinical aspects of the project. TA, AJB, BK, MA, AL and LL contributed to sample collection. TA, JM, BK, AHe, and AJB carried out the experiments. TA, JM, AHe, AHo, MP analyzed the data and contributed to the interpretation of the results. MA, LL, and LA supervised technical aspects the project in Benin. TA wrote the manuscript. All authors contributed to the article and approved the submitted version.
